# MCPH1 Lack of Function Enhances Mitotic Cell Sensitivity Caused by Catalytic Inhibitors of Topo II

**DOI:** 10.3390/genes11040406

**Published:** 2020-04-08

**Authors:** María Arroyo, Antonio Sánchez, Ana Cañuelo, Rosalía F. Heredia-Molina, Eduardo Martínez-Molina, Duncan J. Clarke, Juan Alberto Marchal

**Affiliations:** 1 Department of Experimental Biology, University of Jaén, 23071 Jaén, Spain; marroyo@ujaen.es (M.A.);; 2 Department of Genetics, Cell Biology and Development, University of Minnesota, Minneapolis, MN 55455, USA

**Keywords:** MCPH1, Topoisomerase II, chromosome condensation, chromosome segregation, decatenation checkpoint, ICRF, anaphase errors, mitotic cell death, mitotic catastrophe

## Abstract

The capacity of Topoisomerase II (Topo II) to remove DNA catenations that arise after replication is essential to ensure faithful chromosome segregation. Topo II activity is monitored during G2 by a specific checkpoint pathway that delays entry into mitosis until the chromosomes are properly decatenated. Recently, we demonstrated that the mitotic defects that are characteristic of cells depleted of MCPH1 function, a protein mutated in primary microcephaly, are not a consequence of a weakened G2 decatenation checkpoint response. However, the mitotic defects could be accounted for by a minor defect in the activity of Topo II during G2/M. To test this hypothesis, we have tracked at live single cell resolution the dynamics of mitosis in MCPH1 depleted HeLa cells upon catalytic inhibition of Topo II. Our analyses demonstrate that neither chromosome alignment nor segregation are more susceptible to minor perturbation in decatenation in MCPH1 deficient cells, as compared with control cells. Interestingly, MCPH1 depleted cells were more prone to mitotic cell death when decatenation was perturbed. Furthermore, when the G2 arrest that was induced by catalytic inhibition of Topo II was abrogated by Chk1 inhibition, the incidence of mitotic cell death was also increased. Taken together, our data suggest that the MCPH1 lack of function increases mitotic cell hypersensitivity to the catalytic inhibition of Topo II.

## 1. Introduction

DNA Topoisomerase II (Topo II) is essential in eukaryotic cells due to its capacity to remove the catenations that arise between DNA molecules after replication, a result of the helical conformation of the DNA [1,2]. As catenations physically link sister DNA molecules, they have to be disentangled to allow for proper chromosome condensation and segregation during mitosis [3,4,5]. To release DNA tangles, Topo II performs a multistep process that is known as the strand passage reaction, which includes consecutive conformational changes. This reaction requires the hydrolysis of ATP in order to pass one double stranded DNA molecule through another via a transient double strand break (DSB) [6,7]. The Topo II catalytic cycle has been a major target of multiple anti-cancer compounds due to its crucial role for cell division [1,8]. 

Topo II activity is monitored during the cell cycle by specific checkpoints to ensure the fidelity of cell division. One of these pathways acts in G2 and delays entry into mitosis until chromosomes are properly decatenated [9]. This checkpoint—named the G2 decatenation checkpoint—is triggered after the inhibition of Topo II with catalytic inhibitors that trap the Topo II-DNA complex in a closed-clamp conformation, once the cleaved DNA molecule is re-ligated. As a consequence, this G2 pathway is distinct from the G2 DNA damage checkpoint, as it is not triggered in response to double strand breaks (DSBs) [10,11,12,13]. Another catenation-sensitive checkpoint operates during mitosis and it delays the metaphase-anaphase transition in response to residual chromosome catenation [2,14]. Interestingly, a functional interplay between each of these Topo II-dependent checkpoints might exist, since an elevated dependency on the mitotic checkpoint has been described for cell lines harboring a weak G2 decatenation checkpoint response [15]. Accordingly, the metaphase decatenation checkpoint pathway is considered to be a failsafe mechanism that responds to catenation persisting in the chromosomes from G2 phase. Of interest, the attenuated status of the G2 decatenation pathway described in some tumor cells could have favored the oncogenic process by inducing chromosome instability phenotypes [16,17,18,19,20]. It is noteworthy that some of these tumor cells are more susceptible to the catalytic inhibition of Topo II with dioxopiperazine derivatives, such as ICRF193 and ICRF187 [18,19]. 

MCPH1 primary microcephaly is a rare genetic syndrome that is characterized by pronounced reduction of the cerebral cortex, mental retardation, and delayed growth [21,22]. It is proposed that neurogenic progenitors are more susceptible than other cell types to mutations in genes, such as MCPH1, which control basic mitotic events [23]. Among the processes that MCPH1 participates in during the cell cycle—e.g. centrosome function, DNA repair, and telomere maintenance (reviewed in [24])—chromosome functions are particularly notable. During unperturbed cell division, MCPH1 regulates the process of chromosome condensation by controlling the loading of condensin II complex into the chromatin. Consequently, the period of the cell cycle during which chromosomes are condensed is elongated in MCPH1 depleted cells, since condensation is prematurely initiated in mid-G2 phase and chromosomes remain condensed during the next G1-phase of the cell cycle [25,26,27]. The structure of mitotic chromosomes is also perturbed in cells lacking MCPH1 function. In particular, chromosomes are hypercondensed and the sister chromatids frequently appear unresolved and have a hypercoiled appearance [28]. Furthermore, MCPH1 function is required for efficient chromosome biorientation on the mitotic spindle during prometaphase [29]. Thus, MCPH1 depleted cells require more time to align all of the chromosomes at the metaphase plate when compared with the control cells. This results in a mitotic delay. Interestingly, anaphase errors, such as bridged or lagging chromosomes, are slightly increased when the MCPH1 function is compromised [29].

The mitotic defects summarized above could indicate that decatenation activity is deficient in cells lacking MCPH1 function. Consistent with this hypothesis, incomplete decatenation is one of the main mechanisms underlying lagging or bridged chromosomes [15,30,31]. However, our recent data demonstrated that the G2 decatenation checkpoint is functional in MCPH1 depleted cells. In fact, the duration of G2 arrest that is induced by catalytic inhibition of Topo II is longer in MCPH1 depleted cells when compared to control cells [13]. This leaves open the question, what is the molecular basis of the mitotic defects that arise after MCPH1 depletion, and to what extent might they be a consequence of perturbed decatenation. An emerging question is whether the mitotic defects arise due to a minor defect in the activity of Topo II during G2/M. In that case, mitotic progression and chromosome segregation would be predicted to be more strongly affected by perturbations of Topo II activity. We have tracked at live single cell resolution the dynamics of mitosis in MCPH1 depleted cells under conditions where Topo II activity is compromised to gain further insight into this question. 

## 2. Materials and Methods

### 2.1. Ethic Statement

This study was approved by “Comisión de Ética”, Vicerrectorado de Investigación, Universidad de Jaén, Spain, under the reference number OMGsABs-385.

### 2.2. Cell Culture and Treatments

We have used a modified HeLa cell line—H2B/Red1 and alpha-tubulin/GFP tagged which was originally established at Duncan J Clarke’s laboratory [29]. Cell culture was done following standard conditions, in a humidified atmosphere under 5% CO_2_ at 37 °C while using DMEM medium that was supplemented with 10% of fetal bovine serum (FBS) and antibiotics (1% Penicillin-Streptomycin). The cells were split every two or three days. For RNA interference (RNAi) treatments, cells were transfected with 120 nM small interfering RNA (siRNA) duplexes using Lipofectamine (Invitrogen; ThermoFisher Scientific, Waltham, MA, USA) at 75% confluence and they were imaged live or processed approximately 24 h after transfection. OptiMEM medium (Invitrogen) was used for cell transfection. The MCPH1_siRNA duplexes used are based on previous studies and knocked-down the MCPH1 protein levels efficiently [13,29,32]. MCPH1 siRNAs were purchased from Qiagen (Germantown, MD, USA). As a negative control in our experiments, we used scrambled siRNA duplexes (ThermoFisher Scientific). The synchronization of cells at G1/S was achieved by a double-thymidine blocking protocol (final concentration of 2 mM thymidine in medium), as detailed in [13,29]. The inhibitors employed were ICRF-193 and ICRF-187 (final concentration depending on the experiment), CHIR124 (final concentration 150 nM) and etoposide (15 µM) are from Sigma-Aldrich (St. Louis, MO, USA). The untreated control cells were incubated in all cases with a similar volume of inhibitor solvent (dimethyl sulfoxide).

### 2.3. Live-Cell Microscopy

The procedure was similar to the described at [13,29]. The cells were plated at 60% of confluence onto 35 mm four-square tissue culture dishes fitted with glass cover-slips (MatTek Cultureware; Ashland, MA, USA). siRNA transfection and cell synchrony was performed, as described in the results and discussion section, except that upon release from the second thymidine the standard medium containing the thymidine was exchanged for DMEM without phenol red, supplemented with 10% FBS, penicillin/streptomycin, and 200 mM Trolox (Calbiochem; San Diego, CA, USA) prewarmed in incubator at 37 °C. The dishes were transferred to a microscope humidified stage incubator containing 5% CO_2_ at 37 °C. The cells were filmed with three to five *z* sections while using an inverted laser scanning Leica TCS SP5 microscope fitted with 20x objective and zoom 2x and coupled with Confocal LAS AF software (Leica Application Suite for Advanced Fluorescence; Leica Microsystems, Wetzlar, Germany). The cells were imaged live between 4–6 h after thymidine release during the next 16–20 h. The TIFF images were stacked and processed using Image J software (https://imagej.nih.gov/). Timing data were obtained after visual inspection of a minimum of 50 cells. Statistical comparisons were done using Statgraphics software (Statgraphics Technologies, Inc., The Plains, VA, USA). 

### 2.4. Immunofluorescence

For γH2AX inmunofluorescence staining, the cells were seeded on polylysine A–coated glass coverslips, being previously sterilized with UV light. Cells growing on treated glass coverslips were synchronized at the G1/S border by double thymidine block and transfected with siRNAs, as described in the results and discussion sections. ICRF (4-[2-(3,5-Dioxo-1-piperazinyl)-1-methylpropyl]piperazine-2,6-dione) was added 6 h after release from the second thymidine block, and the cells were processed 3 h after. Cells were fixed with 4% paraformaldehyde in 1 x PBS (pH 7.4) for 15 min at room temperature and then permeabilized with ice-cold methanol for 30 min on ice. Cells were incubated with 1 x PBS containing 3% BSA as a blocking agent for 30 min and then with the primary antibody solution containing a final concentration of 0.5% BSA and mouse anti-γH2AX (ref. 05-636, dilution 1:500; MilliporeSigma, Sigma-Aldrich). After being washed three times with 1 x PBS, cells were incubated with secondary antibody solution, 0.5% BSA in 1 x PBS, and anti-mouse IgG AlexaFluor 594 (ref. A32723, dilution 1:500; ThermoFisher Scientific) for 1 h. The cells were finally counterstained with 1 µg/mL DAPI (4´,6- diamidin-2-phenylindol; Sigma-Aldrich) and the coverslips were mounted with MOWIOL (Sigma-Aldrich). The images were acquired with the Operetta system (Perkin Elmer, Beaconsfield, UK). Number of foci per cell (spots per nuclei) were scored using Opperetta high-content screening system and quantified with the Harmony software by the following workflow: cell nuclei were first identified according to their DAPI fluorescence and evaluated for morphological properties, like roundness and size. These parameters, as well as the mean and sum nuclear fluorescence of DAPI, AlexaFluor 594-labelled gH2AX, and the number of nuclear Alexa 594 spots were calculated in cells that fit these morphological criteria. The measurement results from 10.000 cells were processed and plotted with R Studio (Version 1.1.447; https://rstudio.com/)

### 2.5. Western Blot

Approximately 1 x 10^5^ cells were suspended in 100 μl of lysis buffer, sonicated and boiled for 2 min. The proteins were resolved by SDS-PAGE and then transferred to Hybond-P PVDF membranes (Amersham, Little Chalfont, UK). The membrane was blocked with 2.5% (w/v) dry milk in TBS-T (20 mM Tris-HCl (pH 7.5), 150 mM NaCl, 0.05% Tween 20). For detecting phosphoS345-CHK1, dry milk was replaced by BSA in the blocking solution. Incubation with primary antibodies was performed in TBS-T containing 1% BSA and 0.05% sodium azide overnight at 4 ºC. Blots were developed by enhanced chemiluminescence detection system (Amersham). The primary antibodies used were anti-MCPH1 (11962-1-AP, dilution 1:500; Proteintech, Manchester, UK), anti-phosphoS345-Chk1 (13323 dilution 1:500; Cell Signaling), and anti-alpha tubulin (ref. T5168, dilution 1:1000; Sigma-Aldrich) as the loading control. Relative quantification of phospho-Chk1 protein levels upon normalization with loading control was done with the ImageJ software. 

## 3. Results and Discussion

**Figure 1:**
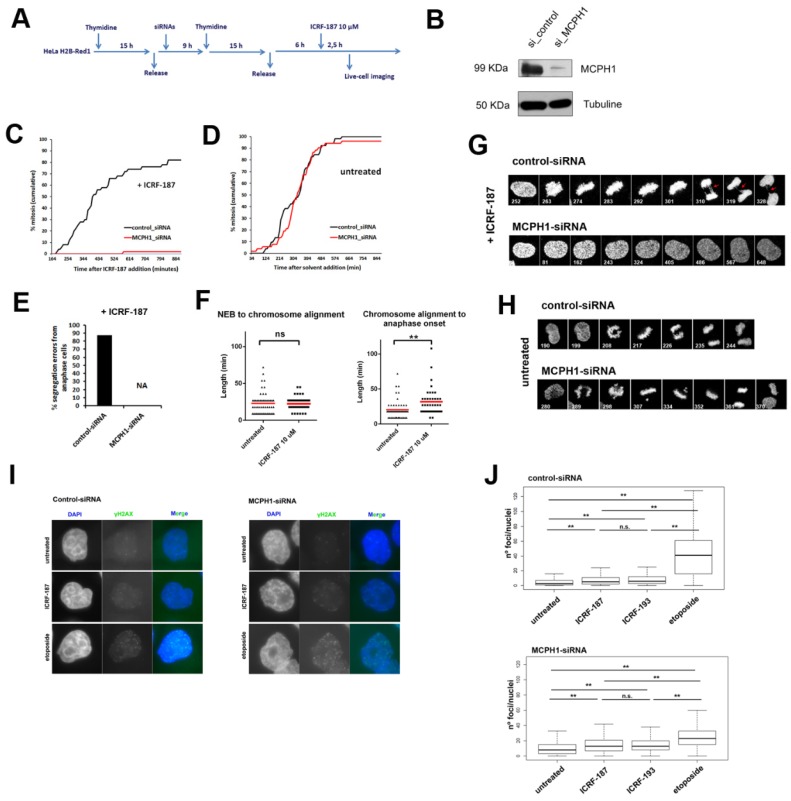
Catalytic inhibition of Topo II by ICRF induces permanent G2 arrest in MCPH1 depleted cells in the absence of double strand breaks (DSBs). (**A**) Description of the experimental procedure performed in HeLa cells stably expressing fluorescent H2B-Red1 and αTubuline-GFP. Cells were synchronized at the G1/S border by double thymidine block. Transfection with small interfering RNAs (siRNAs) duplexes was performed during the release from the first thymidine block. ICRF-187 (10 µM) was added 6 h after release from the second thymidine block to coincide with the occurrence of PLCs (“Prophase-like” cells) during G2 in the siRNA treated cells [1]. Fluorescent images were collected immediately after release from ICRF-193 incubation (2 h) with a Leica TCS SP5 microscope. Images were stacked and processed using Image J software. Timing data were obtained after visual inspection of a minimum of 50 cells. (**B**) Immunoblot analyses of MCPH1 and alpha-tubulin (loading control) levels in HeLa H2B-Red1 cells treated as explained in A. (**C**, **D**) Cumulative frequency chart showing the timing (in min) of mitosis onset, revealed by nuclear envelope breakdown, for cells monitored as explained in A. Time after ICRF-187 or solvent addition is shown. Data representative of two independent experiments is shown. (**E**) Frequency of segregation errors for cells in anaphase from A (*n* = 50). Data from MCPH1-siRNA samples were not analyzed as no mitotic cells were observed (NA, not analyzed). (**F**) Box-plots showing the time interval of the indicated mitotic events in min. for the indicated treatments. The red line indicates the mean value. At least 50 cells were analyzed in each case. Statistical comparisons for the mean and median data were performed by *t*-student and Wilcoxon (*W*) tests respectively. ** *p* < 0.01; N.S. = not significant. (**G, H**) Selected frames showing the cell cycle dynamics of representative control-siRNA and MCPH1-siRNA transfected cells upon the indicated treatments. Red arrows point to bridge figures persisting during late mitosis in control cells. Time from onset of live-cell recording is indicated in min. (**I**) Representative images of mid-Z sections from cells after 3 h of incubation with ICRF-187 (10 uM), etoposide (15 uM) or mock. (**J**) Box-plots showing the number of γ-H2AX foci per nucleus observed by immunofluorescence after 3 h of incubation with the corresponding inhibitors. Boxplots represent the median with the box depicting the 25–75 percentile and the lines denote the 95% confidence interval. Statistical significance was tested with a paired two-samples Wilcoxon test, ** *p* < 0.005, N.S. not significant. NEB: Nuclear Envelope Breakdown.

The aim of this study was to analyze how the inhibition of Topo II activity during G2/M influences the mitotic defects characteristic of cells lacking MCPH1 function. We made use of HeLa cells expressing H2B-Red1 and alpha-tubulin-GFP monitored by live-cell microscopy to achieve this at single-cell level resolution. The depletion of MCPH1 protein was achieved in these cells by RNAi using previously characterized protocols [13,29] (Figure 1B). Transfection with siRNAs was coupled with cell synchronization at G1/S using an excess of thymidine. ICRF-187, a catalytic inhibitor of Topo II, was added 6 h after release from the second thymidine arrest. Using this protocol, Topo II activity is blocked once the cells are at mid-G2 phase, avoiding indirect effects due to interference with S-phase completion [29] (experimental outline in Figure 1A). Live-cell imaging was initiated 2.5 h after Topo II inhibition. The control cells were able to adapt to the decatenation checkpoint arrest induced by ICRF-187 and progressively accumulated in mitosis, as depicted in Figure 1C. As expected from the lack of functional Topo II, most of these cells exhibited chromosome segregation defects, such as lagging or bridged chromosomes (Figure 1E,G). Segregation errors are the expected outcome for cells that are deprived of decatenation during G2 and mitotic progression. In relation to this, a mitotic checkpoint that responds to chromosome catenation operates during metaphase, causing delayed anaphase onset [14,15]. This mitotic checkpoint response was observed in control cells after Topo II inhibition, since metaphase duration was significantly increased (31.8 ± 19.3 min) when compared with untreated cells (20.4 ± 13.2 min) (Figure 1F). By contrast, the time spent in prometaphase was not altered in ICRF-treated control cells (22.5 ± 9.3 min.) compared with untreated cells (23 ± 14.8 min), in line with previous reports [15] (Figure 1F).

**Figure 2:**
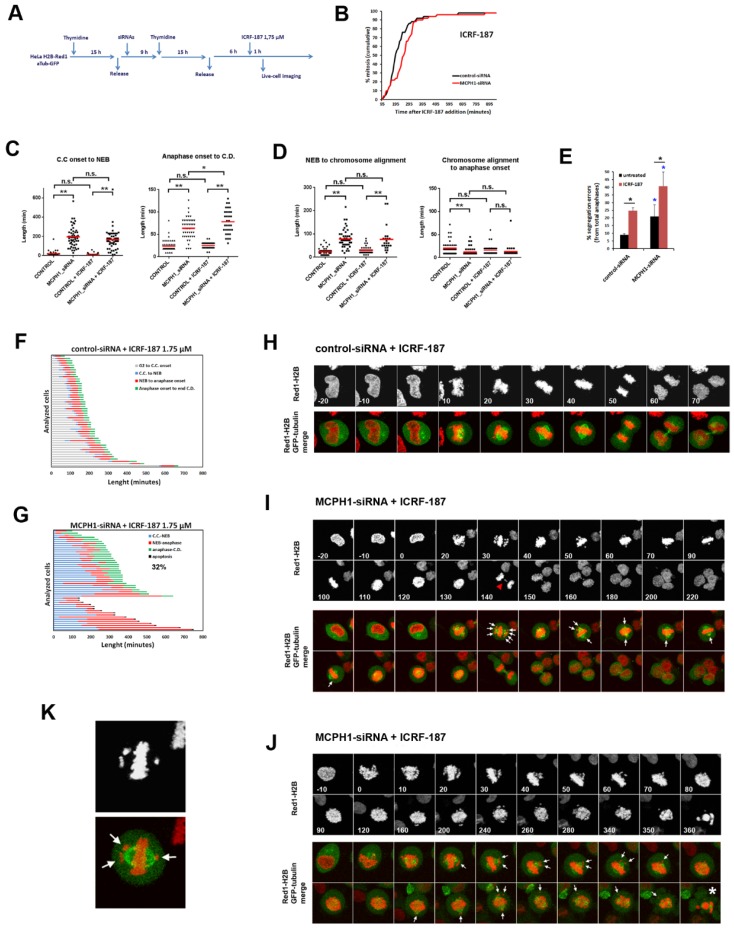
Low dose inhibition of Topo II has a negative impact on the viability of mitotic cells lacking MCPH1 function. (**A**) Description of the experimental procedure performed. (**B**) Cumulative frequency chart showing the timing (in min) of mitosis onset, revealed by nuclear envelope breakdown, for 50 cells monitored as explained in A. Time after ICRF addition is shown. Data representative of two independent experiments is shown. (**C, D**) Box-plots showing the time interval of the indicated mitotic events in min. for the indicated treatments. The red line indicates the mean value. 50 cells were analyzed in each case. Statistical comparisons for the mean and median data were performed by *t*-student and Wilcoxon (*W*) tests respectively. **p < 0.01; N.S. not significant. (**E**) Frequency of segregation errors for control-siRNA and MCPH1-siRNA after the corresponding treatments. Mean and range (bars) data from two independent experiments are presented. Pooled data were pairwise compared by Χ2 test of independence. *p < 0.05. (**F, G**) Dynamics of G2/M progression for individual cells analyzed in A. The timing of different key mitotic events is shown in different colors. The number refers to the percentage of cells undergoing mitotic cell death. (**H, J**) Selected frames showing the mitotic progression of representative control-siRNA and MCPH1-siRNA treated cells as explained in A. Red arrowhead points to lagging chromosomes in anaphase. White arrows point to unaligned chromosomes. Asterisk denotes the occurrence of mitotic cell death, which was defined by massive chromosome shattering. Time in min. from nuclear envelope breakdown (0 min) is shown. (**K**) Illustrative example of a prometaphase cell depleted of MCPH1 function. Note that unaligned chromosomes, indicated by arrows, appear close to the spindle poles. C.C: Chromosome condensation, C.D.: Chromosome decondensation.

Our approach proved to be appropriate to track the dynamics of mitosis at single-cell level resolution upon catalytic inhibition of Topo II. However, upon ICRF-187 incubation, MCPH1 depleted cells remain permanently arrested in G2 and no spontaneous checkpoint bypass is observed (Figure 1C). These results are similar to those that were previously described using ICRF-193, a related bisdioxopiperazine derivate [13]. MCPH1 depleted cells remained permanently arrested in G2, but with visible chromosome condensation inside the intact nucleus. This hypercondensed state in G2, which is a cellular hallmark of MCPH1 syndrome [29,33], was progressively reversed upon prolonged incubation with ICRF-187 (Figure 1G). Therefore, in MCPH1 depleted cells, ICRF-193 and ICRF-187 induced G2 cell cycle arrest and the prematurely condensed chromosomes typical of MCPH1 deficiency then became decondensed (unlike in untreated MCPH1 depleted cells, where the chromosomes remained condensed in the G2-arrested cells). In the absence of Topo II inhibition, control and MCPH1-siRNA treated cells both showed similar timing of entry into mitosis (Figure 1D). However, the MCPH1 depleted cells took longer to align the chromosomes on the metaphase plate during prometaphase, which indicated the successful depletion of MCPH1 protein levels (Figure 1H and Figure 2F; [29]).

Consistent with previous studies (e.g. [13]), we observed a minor increase in the frequency of γH2AX foci upon incubation with ICRF-187 or ICRF-193 at high doses in both control—and MCPH1 depleted cells. Therefore, the Topo II catalytic inhibitors do not induce a large number of DSBs in HeLa cells. By comparison, Etoposide, a Topo II poison that generates DSBs after trapping the covalent enzyme-DNA complex, generated far more DSBs (Figure 1I,J). The MCPH1 depleted cells remained permanently arrested in G2 despite the minor increase in γH2AX foci upon ICRF-187 incubation in both control and MCPH1 depleted cells, whereas the control cells entered mitosis after only a short delay (Figure 1C). Importantly, while MCPH1 function is essential for adaptation to the ICRF-induced G2 arrest, it is not required to bypass an etoposide-induced G2 arrest [13]. Therefore, ICRF incubation in G2-phase triggered a cellular response that is not due to DSBs [9,12,13,20,34]. In addition, our analyses demonstrated that chromosome segregation is compromised in HeLa cells when decatenation is perturbed by ICRF treatment during G2/M progression.

We repeated the experiment described above with a reduced dose of inhibitor to avoid the permanent G2 arrest that catalytic inhibition of Topo II induces in MCPH1 depleted cells. In this way, we aimed to reduce the decatenation capacity of the cells, but not to an extent that caused a prolonged G2 checkpoint arrest. Using this strategy (summarized in Figure 2A), cells that were depleted of MCPH1 responded similarly to control cells, progressing into mitosis upon incubation with the low dose of ICRF-187 (Figure 2B; Video S1 and Video S2). Therefore, the ability of MCPH1 depleted cells to bypass the G2 decatenation checkpoint is dependent on the dose of Topo II inhibitor. Despite the low ICRF-187 concentration that was used in our experiment, the decatenation capacity of cells was compromised because the frequency of chromosome segregation errors was increased in both ICRF-treated control and MCPH1-depleted cells as compared with the untreated paired samples (Figure 2E,I). Together, the data indicate that Topo II activity was partially compromised under our experimental conditions. However, while the basal level of anaphase errors was higher in cells lacking MCPH1 function during unperturbed cell cycle progression, in line with previous reports [29], upon Topo II inhibition, the fold increase in anaphase errors was not higher in the MCPH1-siRNA treated cells (1.9 fold) as compared to control-siRNA treated cells (2.7 fold) (Figure 2E). Therefore, the error-prone segregation induced when the activity of Topo II was partially inhibited is not aggravated by the lack of MCPH1 function.

Interestingly, our analyses revealed that MCPH1 depleted cells are more susceptible to mitotic cell death under these conditions. Indeed, 33% of the analyzed cells underwent mitotic death (as visualized by shattering of the chromosomes in the live-cell movies; Figure 2G and an illustrative example in Figure 2J) before attempting chromosome segregation. These cells remained arrested in a prometaphase state, being characterized by most of the chromosomes being aligned at the metaphase plate, while a few chromosomes remained unaligned and located apparently out of the spindle (Figure 2J,K; Video S3). These phenotypes were never observed in the control-siRNA treated cells, which were always able to progress into anaphase and successfully finish mitosis (Figure 2F; illustrative example in Figure 2H; Video S1). 

The MCPH1 depleted cells that were able to finish mitosis required more time to decondense their chromosomes once segregation was achieved when compared with control cells. Thus, chromosome condensation remained visible after segregation for 78 ± 27 min in MCPH1 cells while in control cells it was observed for only 25 ± 6 min (Figure 2C). Similarly, chromosome condensation was prematurely initiated (in G2) and the chromosomes remained in this state for 165 ± 15 min before NEB in MCPH1-siRNA treated cells, while in control cells NEB occurred 14 ± 9 min after the initiation of chromosome condensation (Figure 2C). These results are indicative of the efficient knock-down of MCPH1 protein levels, since premature chromosome condensation and delayed decondensation are cellular hallmarks of MCPH1 lack of function [29,33]. Importantly, the timing of the initiation of condensation before NEB and decondensation after chromosome segregation in both ICRF-treated control and MCPH1 depleted cells remained comparable to the untreated paired samples (Figure 2C). This result indicates that the altered condensation dynamics that are characteristic of MCPH1 depleted cells are not affected by mild Topo II inhibition.

Next, we asked whether the prometaphase delay characteristic of MCPH1 depleted cells [29] is influenced by partial inhibition of Topo II. From the MCPH1-depleted, ICRF-treated cells that were able to complete mitosis, we determined the duration of prometaphase and compared this to MCPH1-depleted cells not treated with the Topo II inhibitor. The time that MCPH1-depleted cells required to achieve full chromosome alignment at the metaphase plate after ICRF treatment was similar to that observed without the Topo II inhibitor, as shown in Figure 2D (76 ± 55 min and 78 ± 55, respectively). While most of the chromosomes rapidly bioriented at the metaphase plate, some chromosomes remained unaligned for a long time, as depicted in Figure 2I. The unaligned chromosomes appeared to be located out of the equatorial plane and they were frequently close to the spindle poles (see illustrative example in Figure 2K). Therefore, in cells lacking MCPH1 function, the process of chromosome biorientation is not lengthened if Topo II activity is partially compromised. It should also be noted that the level of Topo II inhibition achieved was not high enough to trigger the metaphase decatenation checkpoint response, since no metaphase arrest was observed either in control cells or in MCPH1 depleted cells after ICRF treatment when compared with the untreated paired samples (Figure 2D).

**Figure 3:**
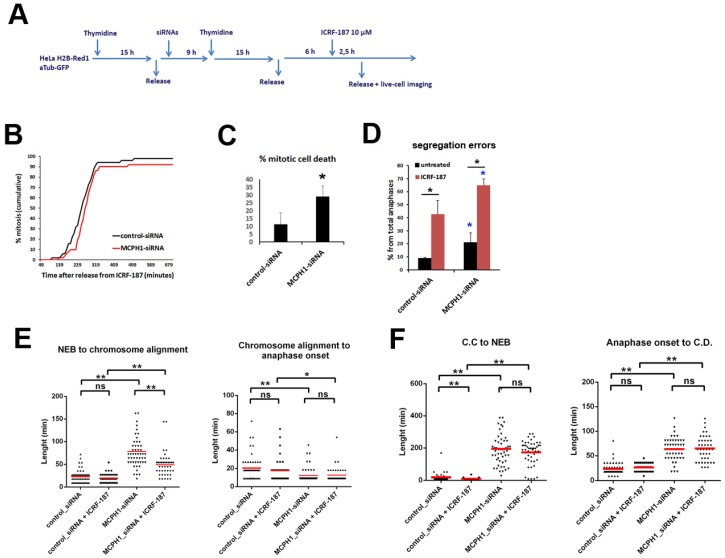
Upon recovery from ICRF187-mediated G2 arrest MCPH1 depleted cells have reduced viability during mitosis. (**A**) Description of the experimental procedure performed. (**B**) Cumulative frequency chart showing the timing (in min) of mitosis onset, revealed by nuclear envelope breakdown, for 50 cells monitored as explained in A. Time after release from ICRF-187 addition is shown. Data representative of two independent experiments is presented. (**C, D**) Charts showing the frequency of mitotic cell death (C) and segregation errors during anaphase (D) from the cells analyzed in A. Mean and range (bars) data from two independent experiments are presented. Pooled data were pairwise compared by χ^2^ test of independence. *p < 0.05. (**E, F**) Box-plots showing the time interval of the indicated mitotic events in min. for the indicated treatments. The red line indicates the mean value. 50 cells were analyzed in each case. Statistical comparisons for the mean and median data were performed by *t*-student and Wilcoxon (*W*) tests respectively. **p < 0.01

Next, we investigated whether the cellular behavior observed upon partial inhibition of Topo II activity is recapitulated when cells enter mitosis after recovery from prolonged ICRF-induced G2 arrest. We define recovery as the process that restarts cell cycle progression once the Topo II catalytic inhibitor is removed. To address this, we used live-cell imaging immediately after the release from incubation with ICRF-187, which was added during mid G2 phase in order to trigger the decatenation checkpoint (experimental outline in Figure 3A). First, the results demonstrate that the MCPH1 depleted cells have the capacity to resume cell cycle progression after recovery from prolonged incubation with ICRF-187 at high concentration (Figure 3B). Next, we observed an increased frequency of mitotic cell death in MCPH1 depleted cells upon recovery from Topo II inhibition as compared with control cells. Therefore, on average, 29% of cells underwent mitotic death before being able to initiate chromosome segregation (Figure 3C). In addition, the frequency of segregation errors was increased in both control and MCPH1 depleted cells upon recovery from ICRF-187 incubation. However, when we determined the fold increase in the frequency of segregation errors when compared with cells that were not treated with ICRF, it was not higher in MCPH1 depleted cells (three-fold increase on average) than in the control cells (4.7-fold increase on average) (Figure 3D). Finally, the timing of condensation before NEB and decondensation after chromosome segregation in the control and MCPH1 depleted cells upon recovery remained comparable to paired samples not treated with ICRF (Figure 3F). Together, these results are in line with previous observations of cells progressing through mitosis in the presence of the low dose of the Topo II inhibitor (Figure 2C,E). 

We also compared the duration of prometaphase and metaphase for the cells that completed mitosis in these recovery experiment. Similar to the previous findings (Figure 2D), the MCPH1 depleted cells did not require more time to align all the chromosomes at the metaphase plate after recovery from ICRF-187 incubation (50 ± 27 min) compared with untreated MCPH1 depleted cells (78 ± 38 min) (Figure 2E). Moreover, the duration of metaphase was not altered as a consequence of the recovery, either in control or in MCPH1 depleted cells (Figure 3E), again in line with the previous findings (Figure 2F). Similar results were observed when ICRF-193 was used for the recovery analyses (data not shown). 

Taken together, the results show that the increased frequency of mitotic cell death that was observed in MCPH1 depleted cells with the low dose of Topo II inhibitor was also evident when these cells recovered from ICRF-induced G2 arrest. However, in both scenarios, the processes of chromosome alignment and segregation were perturbed to a similar extent in MCPH1 depleted and control cells, when compared with cells that were not treated with ICRF. The extended prometaphase characteristic of MCPH1 depleted cells was not lengthened by treatment with ICRF, and the fold increase in anaphase errors was similar in the control and MCPH1 depleted cells with and without ICRF. Therefore, it seems that neither alignment nor chromosome segregation are more susceptible to minor decatenation perturbations in cells lacking MCPH1 function as compared with control cells. 

**Figure 4:**
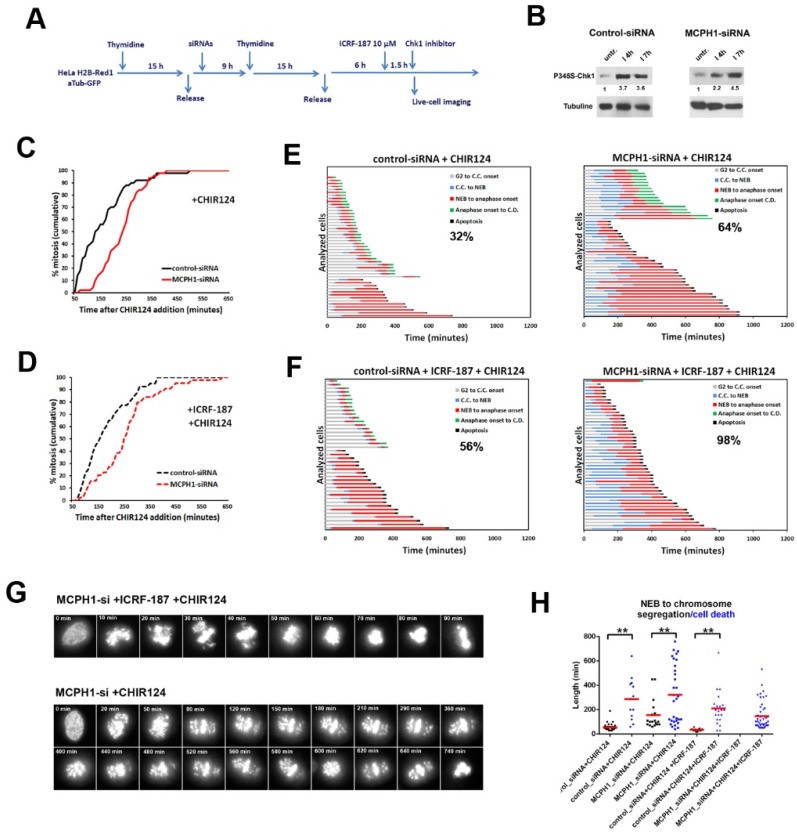
Cells lacking MCPH1 function are hypersensitive to abrogation of the ICRF187-induced G2 arrest by Chk1 inhibition. (**A**) Description of the experimental procedure performed. (**B**) Immunoblot analyses of phosphoS345-Chk1 and tubulin levels in control-siRNA and MCPH1-siRNA cells after incubation with ICRF-193 for the indicated times. Relative comparison of phosphoS345-Chk1 levels upon normalization with loading control is shown below each sample. (**C, D**) Cumulative frequency chart showing the timing (in min) of mitosis onset, revealed by nuclear envelope breakdown, for 50 cells monitored as explained in A. Time after CHIR124 or solvent addition is shown. Data representative of two independent experiments is shown. (**E, F**) Dynamics of G2/M progression for individual cells analyzed in A. The timing of different key mitotic events is showed in different colors. The number refers to the percentage of cells undergoing mitotic cell death in each sample. (**G**) Selected frames showing MCPH1-siRNA cells undergoing mitotic cell death upon the indicated treatments. Time in min. from nuclear envelope breakdown (0 min) is shown (**H**) Box-plots showing pairwise comparisons of the time that cells within the same sample (indicated) required to either initiate mitotic cell death (black symbols) or initiate chromosome segregation (blue symbols), after nuclear envelope breakdown. Red lines denote the mean value. Statistical comparisons for the mean and median data were done by *t*-student and Wilcoxon (*W*) tests respectively. ** *p* < 0.01

Finally, we asked whether the MCPH1 depleted cells are more prone to mitotic death when dividing in the presence of the high dose of Topo II inhibitor. To test this, it was necessary to avoid the permanent G2 arrest induced by ICRF-187 when added at high dose in MCPH1 depleted cells. Recent data confirmed that the Chk1 function is required to sustain the ICRF-193-induced G2 arrest in human cells [35]. Accordingly, the levels of Chk1 phosphorylated at Ser345, a hallmark of Chk1 activation (30, 31), are increased upon prolonged incubation with a high dose of ICRF-193 in both control and MCPH1 depleted cells (Figure 4B). In addition, Chk1 inactivation restores the capacity of MCPH1 depleted cells to bypass the G2 decatenation checkpoint arrest [35]. Therefore, we made use of a highly specific and potent Chk1 inhibitor, CHIR124 [36], in order to override the permanent G2 arrest induced by ICRF in these cells. CHIR124 was added two hours after ICRF-187 following the procedure that is described in Figure 4A. ICRF187-treated control and MCPH1 depleted cells both progressed into mitosis when incubated with CHIR-124, as depicted in Figure 4D; only a minor delay was observed for MCPH1 depleted cells compared with control cells. This delay was also observed in MCPH1 depleted cells when the same analyses were repeated, but without adding ICRF-187, which suggested that it is a direct consequence of inactivating Chk1 in these cells (Figure 4C). 

Our analyses revealed that bypass of the ICRF187-induced G2 arrest by CHIR124 treatment triggered a higher level of mitotic cell death in MCPH1 depleted cells as compared with control cells (Figure 4F). Strikingly, 98% of MCPH1 depleted cells underwent mitotic cell death in metaphase, before being able to initiate chromosome segregation. By contrast, 44% of control cells successfully completed mitosis under the same conditions (Figure 4F). A high incidence of mitotic cell death was also observed in MCPH1 depleted cells that were treated with CHIR24 in combination with ICRF-193 (data not shown). In addition, MCPH1 depleted cells were more sensitive to single incubation with CHIR124 when compared with control cells. As shown in Figure 4E, 64% of MCPH1 depleted cells underwent mitotic cell death in with CHIR124 treatment, while in the control cells the frequency was reduced to 32% under the same conditions. In general, cells that underwent mitotic cell death remained arrested in prometaphase or metaphase for longer times as compared with those successfully completing mitosis within the same sample (Figure 4G). 

We conclude that HeLa cells that are depleted of MCPH1 are more sensitive to the inhibition of Chk1. The identification of genetic factors that trigger hypersensitivity to Chk1 inhibition and induce mitotic cell death is of interest because a subset of cancer cells—including small cell lung cancer and colorectal cancer cells—are particularly sensitive to the single inhibition of Chk1 [37]. Furthermore, we demonstrate here that the abrogation of the ICRF187-induced G2 arrest with CHIR124 has a lethal effect in HeLa cells depleted of MCPH1 in mitosis. In contrast, many control cells retained the capacity to complete mitosis under the same conditions. Previous studies have shown that mitotic cell death is the expected outcome in a variety of human cell lines, when (i) damaged in G2 by agents that induce DSBs in the DNA and (ii) forced into mitosis by Chk1 inhibition [36,38,39]. Our results must be placed in a different context, because catalytic inhibition of Topo II triggers the G2 decatenation checkpoint that does not respond to DSBs, but still requires Chk1 activity [13,35,40]. In this context, MCPH1 is one genetic factor that strongly impacts the viability of mitotic cells after decatenation is perturbed.

An established idea is that mitotic cell death is a mechanism that aims to prevent further genome instability in cells that are defective in DNA damage checkpoints [38]. However, the mechanisms that are involved in mitotic cell death upon G2 checkpoint abrogation are poorly understood. Therefore, mitotic cell death, which is also often referred to as ‘‘mitotic catastrophe’’, is ill defined and both apoptotic and non-apoptotic mechanisms might contribute to this particular form of cell death [41]. Interestingly, previous studies highlighted mitotic catastrophe during neurogenesis as a mechanism that arises upon prolonged mitosis and that might contribute to some microcephaly syndromes [42,43]. Since MCPH1 mutations prolonged the duration of mitosis in non-neurogenic cell populations [29], it will be of interest to further investigate whether cell fate during neurogenesis is correlated with the length of mitosis in MCPH1 models. 

## Supplementary Materials

The following are available online at https://www.mdpi.com/2073-4425/11/4/406/s1, Video S1: HeLa cells stably expressing fluorescent histone H2B-Red1 and αTubuline-GFP after transfected with control-siRNAs and treated with ICRF-187, Video S2: HeLa cells stably expressing fluorescent histone H2B-Red1 and αTubuline-GFP transfected with MCPH1-siRNAs and treated with ICRF-187, Video S3: HeLa cells stably expressing fluorescent histone H2B-Red1 and αTubuline-GFP transfected with MCPH1-siRNAs and treated with ICRF-187.
