# Supplementary material for: MCPH1 Lack of Function Enhances Mitotic Cell Sensitivity Caused by Catalytic Inhibitors of Topo II
Source: Genes (Basel). 2020 Apr 8;11(4):406. doi: 10.3390/genes11040406 (PMC7231051; doi:10.3390/genes11040406)
Supplement: Supplementary file 1 [file genes-11-00406-s001.zip › captions.docx]

**SUPPLEMENTARY MATERIAL**

**Supplementary 1:** video showing HeLa cells stably expressing fluorescent histone H2B-Red1 and αTubuline-GFP after transfected with control-siRNAs and treated with ICRF-187 as explained in figure 2. Single red (right) and merge channels (left) are simultaneously shown. Time after ICRF adding (in minutes) is indicated.

**Supplementary 2:** video showing HeLa cells stably expressing fluorescent histone H2B-Red1 and αTubuline-GFP transfected with MCPH1-siRNAs and treated with ICRF-187 as explained in figure 2. Single red (right) and merge channels (left) are simultaneously shown. Time after ICRF adding (in minutes) is indicated.

**Supplementary 3:** video showing HeLa cells stably expressing fluorescent histone H2B-Red1 and αTubuline-GFP transfected with MCPH1-siRNAs and treated with ICRF-187 as explained in figure 2. Single red (right) and merge channels (left) are simultaneously shown. Time after ICRF adding (in minutes) is indicated.
