# Supplementary figures and images for: MCPH1 Lack of Function Enhances Mitotic Cell Sensitivity Caused by Catalytic Inhibitors of Topo II
Source: Genes (Basel). 2020 Apr 8;11(4):406. doi: 10.3390/genes11040406 (PMC7231051; doi:10.3390/genes11040406)

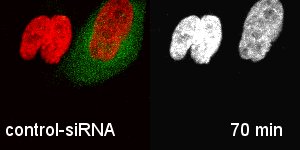

Supplement: Supplementary file 1 [file genes-11-00406-s001.zip › Video1_control.gif]

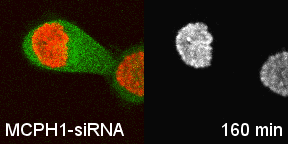

Supplement: Supplementary file 1 [file genes-11-00406-s001.zip › Video2-MCPH1.gif]

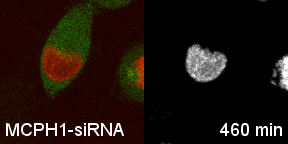

Supplement: Supplementary file 1 [file genes-11-00406-s001.zip › Video3-MCPH1.gif]
